# A Case of Hypercalcemia and Acute Kidney Injury Caused by Adrenal Insufficiency

**DOI:** 10.7759/cureus.74109

**Published:** 2024-11-20

**Authors:** Norihito Yoshida, Yusuke Suzuki, Mai Hitaka, Keisuke Yamazaki, Yasushi Ohashi

**Affiliations:** 1 Department of Nephrology, Toho University Medical Center, Sakura Hospital, Sakura, JPN

**Keywords:** adrenal insufficiency, aki, altered mental, chronic kidney disease (ckd), glucocorticoid supplement, hypercalcemia

## Abstract

Hypercalcemia is a common electrolyte disturbance, most frequently caused by hyperparathyroidism or malignancy, though it can also arise from adrenal insufficiency, creating diagnostic challenges. We present a case of a 78-year-old male patient with stage 3b chronic kidney disease due to immunoglobulin A nephropathy who exhibited altered mental status following dehydration caused by a five-day episode of diarrhea. The patient presented with hypercalcemia and acute kidney injury. His history of adrenal insufficiency had been managed with glucocorticoid replacement. Dehydration and inability to take oral medications led to exacerbation of adrenal insufficiency, worsening his hypercalcemia. Routine causes of hypercalcemia, such as hyperparathyroidism and malignancy, were ruled out. Treatment with intravenous prednisolone and fluid replacement gradually improved serum calcium and mental status, with normalization by the seventh hospital day. This case highlights the importance of timely glucocorticoid therapy in managing adrenal insufficiency-related hypercalcemia and the complex mechanisms involved, including decreased glomerular filtration rate and increased calcium reabsorption.

## Introduction

Primary hyperparathyroidism and malignancy cause approximately 90% of hypercalcemia cases, but less common causes, including adrenal insufficiency, should also be considered. Although adrenal insufficiency is an uncommon cause, it can occasionally present with hypercalcemia, complicating diagnosis and management. Reports indicate that the prevalence of hypercalcemia in Addison's disease ranges from 5.5% to 6% [1-3]. Early recognition is critical, as adrenal insufficiency may present similarly to conditions like acute kidney injury (AKI), leading to diagnostic delays and poorer outcomes if untreated. This case highlights the need to consider adrenal insufficiency in the differential diagnosis of hypercalcemia, especially when common causes are excluded.

## Case presentation

A 78-year-old male patient with stage 3b chronic kidney disease (CKD) secondary to immunoglobulin A nephropathy, with a baseline estimated glomerular filtration rate of 45 mL/minute/1.73 m² and serum creatinine (Cr) of 1.22 mg/dL, adrenal insufficiency, hypertension, previous hepatitis C infection, and lacunar infarction, was followed in our clinic. Two weeks before admission, he experienced diarrhea lasting for five days and was diagnosed with infectious enteritis at the emergency department, where he was prescribed probiotics and subsequently discharged. Although he routinely took hydrocortisone at a dose of 15 mg per day, his appetite did not return following the enteritis. Over time, he grew progressively weaker, making it difficult for him to take his medications and eventually rendering daily activities challenging. The exact duration of missed hydrocortisone doses remains unclear due to his living alone and an uncertain recollection. He was subsequently brought to the emergency room by ambulance. His social history included past tobacco use and regular consumption of 350 mL of beer per day.

On physical examination, his height was 160 cm, weight was 54 kg, and Glasgow Coma Scale score was 13. His vital signs were as follows: temperature, 36.7°C, blood pressure, 115/78 mmHg, heart rate, 110 beats per minute, respiratory rate, 20 breaths per minute, and oxygen saturation, 98% on room air. He exhibited pallor of the palpebral conjunctiva but no scleral icterus. Oral examination revealed dental caries and dryness of the tongue. There was no cervical lymphadenopathy or pharyngeal erythema. Breath sounds were clear bilaterally, and no cardiac murmurs were auscultated. The abdomen was flat and soft, without tenderness, and no edema was noted in the lower extremities, which appeared dry. There was no costovertebral angle tenderness or spine tenderness, and no skin hyperpigmentation was observed. Table 1 displays the blood and urinary test results.

**Table 1:** Blood and urinary test results ACTH: adrenocorticotropic hormone; AG: anion gap; ALP: alkaline phosphatase; ALT: alanine aminotransferase; AST: aspartate aminotransferase; BE: base excess; BUN: blood urea nitrogen; CK: creatine kinase; CRP: C-reactive protein; eGFR: estimated glomerular filtration rate; IgA: immunoglobulin A; IgG: immunoglobulin G; IgM: immunoglobulin M; IU: international units; LDH: lactate dehydrogenase; NAG: N-acetyl-β-D-glucosaminidase; pCO_2_: partial pressure of carbon dioxide; pH: hydrogen ion concentration; pO_2_: partial pressure of oxygen; PTH: parathyroid hormone; PTHrP: parathyroid hormone-related peptide; TP: total protein; WBC: white blood cells; β2MG: beta-2-microglobulin; γ-GTP: gamma-glutamyl transpeptidase

Blood tests	Results	Standard ranges
WBCs	5,670/μL	3,300-9,000/μL
Hemoglobin	10.3 g/dL	13.5-17.5 g/dL
Platelet	25.6 × 104 μL	14-34 × 104 μL
CRP	5.98 mg/dL	≤0.30 mg/dL
TP	7.8 g/dL	6.7-8.3 g/dL
Albumin	3.2 g/dL	3.8-5.2 g/dL
AST	25 IU/L	10-40 IU/L
ALT	13 IU/L	5-45 IU/L
ALP	56 U/L	38-113 U/L
LDH	155 U/L	124-222 U/L
γ-GTP	64 IU/L	≤80 IU/L
CK	22 mg/dL	60-270 IU/L
Sodium	141 mEq/L	137-147 mEq/L
Potassium	4.8 mEq/L	3.5-5.0 mEq/L
Chloride	108 mEq/L	98-108 mEq/L
BUN	30.3 mg/dL	8-20 mg/dL
Creatinine	3.07 mg/dL	0.61-1.04 mg/dL
eGFR	16 mL/minute/1.73 m²	
Calcium	10.6 mg/dL	8.4-10.4 mg/dL
Corrected calcium	11.2 mg/dL	
Phosphorus	5.4 mg/dL	2.5-4.5 mg/dL
Blood glucose	116 mg/dL	73-109 mg/dL
IgG	1,218 mg/dL	870-1,700 mg/dL
IgA	567 mg/dL	110-410 mg/dL
IgM	40 mg/dL	33-190 mg/dL
ACTH	16.7 pg/mL	7.2-63.3 pg/mL
Cortisol	2.7 µg/dL	3.7-19.4 µg/dL
1α.25(OH)_2_ vitamin D	88 pg/mL	20-60 pg/mL
Intact-PTH	13 pg/mL	10-65 pg/mL
Intact-PTHrP	<1.1 pmol/L	<1.1 pmol/L
Immunoelectrophoresis	Polyclonal IgG	
Arterial blood gas analysis		
pH	7.373	7.350-7.450
pO_2_	109.9 mmHg	100 mmHg
pCO_2_	26.5 mmHg	40 mmHg
Bicarbonate ion concentration	15.1 mmol/L	20.0-26.0 mmol/L
BE	-8.7 mmol/L	-3.0 to 3.0 mmol/L
Lactate	2.13 mmol/L	4-14 mg/dL
AG	17.9 mmol/L	10-14 mmol/L
Urinary tests		
pH	5.5	5.0-8.0
Specific gravity	1.019	1.005-1.030
Urinary protein	2+	-
Urine occult blood	-	-
Urine ketone bodies	1+	-
Urine leukocyte reaction	-	-
Sodium	56 mEq/L	
Potassium	39.5 mEq/L	
Chloride	31 mEq/L	
Calcium	8.7 mg/dL	-
Creatinine	56 mg/dL	-
NAG	52.6 U/L	0.7-11.2 IU/L
β2MG	311 μg/gCr	<200/μg/L

Bacterial cultures were negative. Chest X-ray showed no evidence of pneumonia or cardiomegaly. A head CT scan revealed multiple areas of chronic cerebral infarction. Chest and abdominal CT scans identified gallstones and fatty liver, while the spleen, adrenal glands, and kidneys were normal in morphology. No malignancies were observed in the scanned areas. The electrocardiogram demonstrated a sinus rhythm at 106 bpm, with no ST-segment changes. Transthoracic echocardiography revealed an ejection fraction of 63%, with mild mitral regurgitation and no wall motion abnormalities. Upper gastrointestinal endoscopy showed atrophic gastritis following *Helicobacter pylori* eradication, and colonoscopy revealed no gross lesions.

**Figure 1:**
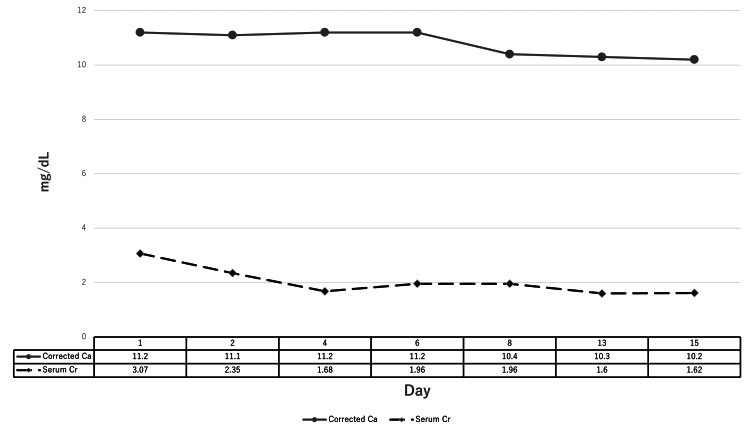
Trends in corrected calcium and serum creatinine levels after hospital admission Serum calcium values are corrected for albumin, with corrected calcium calculated using the following formula: Corrected calcium (mg/dL) = measured total calcium (mg/dL) + 0.8 × (4.0 - serum albumin, g/dL). This adjustment ensures more accurate interpretation of calcium levels in relation to albumin variations

Based on clinical findings and diagnostic imaging, the patient was diagnosed with altered mental status due to multiple factors, including uremia from AKI, adrenal insufficiency, and hypercalcemia. Head imaging was negative for intracranial pathology, and infectious causes were ruled out. Since the patient had been unable to take oral steroids or maintain adequate hydration, adrenal insufficiency was considered, and intravenous prednisolone (PSL) at 10 mg/day was initiated. Although the kidney injury showed signs of improvement with fluid replacement at 2 L/day, hypercalcemia persisted. The patient's mental status gradually improved; by the third hospital day, his consciousness had returned to normal. By the seventh hospital day, serum calcium levels had normalized, and the PSL dose was reduced to 7.5 mg/day. The patient continued to show no recurrence of altered mental status or hypercalcemia and was discharged home on the 16th hospital day. Two weeks after discharge, the patient was seen in the outpatient clinic, where no recurrence of hypercalcemia was noted. His glucocorticoid therapy was switched to hydrocortisone, and he continues to follow up in the outpatient clinic without any further complications. Figure 1 shows the trends in corrected calcium and serum creatinine levels after hospital admission.

## Discussion

Hypercalcemia is a common electrolyte abnormality encountered in outpatient and inpatient settings [1]. It can range from mild to severe, with many cases being asymptomatic. However, when symptomatic, hypercalcemia may present with nonspecific symptoms such as fatigue, polyuria, dehydration, and constipation. In acute and severe cases, it can lead to altered mental status and arrhythmias [1].

This case highlights the importance of timely glucocorticoid replacement in managing hypercalcemia associated with adrenal insufficiency. The patient presented with hypercalcemia associated with adrenal insufficiency, which developed in the context of glucocorticoid replacement therapy.

Initial laboratory and imaging findings did not suggest common causes of hypercalcemia, such as hyperparathyroidism or malignancy. Furthermore, no thiazide diuretics, vitamin D supplements, or lithium, which are known to induce hypercalcemia, had been used. Although urinary calcium excretion was reduced, the calcium/creatinine clearance ratio was not below 0.01, and there was an unlikely family history of familial hypocalciuric hypercalcemia [1]. These factors, along with the absence of hypercalcemia in previous laboratory results and after treatment, suggest that the decreased calcium excretion was related to CKD rather than familial hypocalciuric hypercalcemia. Additionally, there was no evidence of granulomatous disease or thyroid dysfunction. Given the patient's inability to take glucocorticoid medications, low serum cortisol levels, and elevated adrenocorticotropic hormone, primary adrenal insufficiency was diagnosed.

Previous studies have documented similar instances of hypercalcemia in patients with adrenal insufficiency, supporting our findings [4-6]. The patient also exhibited AKI at the time of admission, and fluid replacement therapy was initiated to correct the kidney injury and increase urinary calcium excretion to improve hypercalcemia. Due to altered mental status and the inability to continue oral steroid therapy, glucocorticoid replacement therapy was also initiated. While renal function improved with early treatment, hypercalcemia persisted, suggesting that the resolution of hypercalcemia associated with adrenal insufficiency may take several days after steroid replacement therapy is started.

The incidence of hypercalcemia in secondary adrenal insufficiency is reported to be between 6.5% and 8.4%, while in primary adrenal insufficiency, it is approximately 5.5%-6% [3,7]. Although the exact mechanism of hypercalcemia in adrenal insufficiency is not fully understood, several pathways are thought to be involved [4]. First, adrenal insufficiency leads to hypovolemia and a subsequent decrease in glomerular filtration rate, which increases calcium reabsorption in the proximal tubule [4]. This mechanism is believed to normalize only after glucocorticoid replacement therapy, as fluid replacement alone is insufficient to reduce calcium reabsorption [8].

Second, 1-α-hydroxylase is the enzyme responsible for converting calcidiol into its active form, calcitriol, in the kidneys, thereby promoting the intestinal absorption of calcium. PSL inhibits 1-α-hydroxylase activity, which can help reduce hypercalcemia [1]. In patients with CKD, however, 1-α-hydroxylase activity is typically reduced, leading to lower levels of 1α,25(OH)₂ vitamin D (active vitamin D) and, consequently, impaired calcium absorption [9]. Despite this, in the present case, elevated 1-α-hydroxylase activity was suspected, contributing to the patient's hypercalcemia even in the absence of vitamin D supplementation. This atypical mechanism suggests a complex interplay between adrenal insufficiency and calcium metabolism in CKD.

Third, adrenal insufficiency may lower stanniocalcin levels, a hormone that helps maintain normal serum calcium by inhibiting bone resorption and reducing calcium release into the blood. In adrenal insufficiency, decreased stanniocalcin may lead to an inability to sufficiently suppress calcium release from bones, contributing to elevated serum calcium levels [6]. Furthermore, bone cells possess glucocorticoid receptors, and physiological levels of glucocorticoids are necessary for the differentiation and maintenance of osteoblasts [10]. Disruption of this balance may lead to increased calcium release from bones, contributing to hypercalcemia [8].

Further research is needed to determine whether factors such as calcium-sensing receptor mutations contribute to hypercalcemia in adrenal insufficiency. Additionally, clinical outcomes may have improved more quickly with hydrocortisone, which has greater mineralocorticoid activity, highlighting a limitation in this case.

This case emphasizes the importance of early recognition and timely treatment of adrenal insufficiency in patients with hypercalcemia. Future studies should focus on optimizing glucocorticoid dosing and timing to improve outcomes in similar cases.

## Conclusions

In cases of hypercalcemia, adrenal insufficiency should be considered as part of the differential diagnosis, particularly in patients with a known history of adrenal insufficiency. The diagnostic process requires excluding other common causes, such as hyperparathyroidism, malignancy, and medications. Treatment involves fluid replacement and glucocorticoid supplementation to address the underlying adrenal insufficiency. Early recognition and timely intervention are essential for effective management and improved patient outcomes.
